# Gut Microbiome-Based Analysis of Lipid A Biosynthesis in Individuals with Autism Spectrum Disorder: An In Silico Evaluation

**DOI:** 10.3390/nu13020688

**Published:** 2021-02-21

**Authors:** Abdulkadir Yusif Maigoro, Soojin Lee

**Affiliations:** Department of Microbiology and Molecular Biology, College of Bioscience and Biotechnology, Chungnam National University, Daejeon 34134, Korea; aymaigoro@gmail.com

**Keywords:** autism spectrum disorder, gut microbiome, Gram-negative bacteria, LPS biosynthesis, in silico analysis

## Abstract

The link between autism spectrum disorder (ASD) and the gut microbiome has received much attention, with special focus on gut–brain-axis immunological imbalances. Gastrointestinal problems are one of the major symptoms of ASD and are thought to be related to immune dysregulation. Therefore, in silico analysis was performed on mined data from 36 individuals with ASD and 21 control subjects, with an emphasis on lipid A endotoxin-producing bacteria and their lipopolysaccharide (LPS) metabolic pathways. Analysis of enzyme distribution among the 15 most abundant genera in both groups revealed that almost all these genera utilized five early-stage enzymes responsible for catalyzing the nine conserved lipid A synthesis steps. However, *Haemophilus* and *Escherichia*, which were significantly more abundant in individuals with ASD than in the control subjects, possess a complete set of essential lipid A synthesis enzymes. Furthermore, the 10 genera with the greatest increase in individuals with ASD showed high potential for producing late-stage lipid A products. Collectively, these results suggested that the synthesis rate of immunogenic LPS end products is likely to increase in individuals with ASD, which may be related to their gastrointestinal symptoms and elevated inflammatory conditions.

## 1. Introduction

Autism spectrum disorder (ASD) is a complex neurological disorder characterized by social impairment, learning disability, and inefficient cognition [1]. Current statistical data show that approximately 2.21% of the adults in the United States are living with ASD [2], while 1 in 59 children aged 8 years have been identified as having ASD [3]. Previous studies have reported that ASD can be caused by a range of factors, i.e., genetic factors [4], comorbid disorders of the central nervous system [5], immune dysregulation, and an aberrant innate immune response against endotoxins [6,7]; however, there is no clear evidence to prove these claims.

Endotoxins, or lipopolysaccharides (LPSs), are composed of lipid A (inner membrane) connected to O-antigen (outer membrane) through core polysaccharides [8]. Most Gram-negative bacteria possess LPSs in their outer membrane; it is found at very high levels in the mammalian gut [9]. Endotoxins are present in all humans; however, the levels of LPS in blood vary and are correlated with neurodegeneration [8]. Such variation depends on the composition of lipid A, which also depends on the bacterial strain, species, and environmental conditions [10,11,12]. Further, structural variations in the lipid A domain are characterized by diverse modification patterns (such as acylation) associated with different immunogenicities [13]. This LPS domain found in lipid A is believed to be responsible for the endotoxic properties associated with LPS owing to recognition by the host Toll-like receptor (TLR) 4 complex [14]. As little as picomolar levels of lipid A are sufficient to induce inflammation in humans via TLR4 and myeloid differentiation factor (MD)-2 activation [15]. However, in human cells, the activation of TLR4 and MD-2 requires the presence of two phosphate and acyloxyacyl groups [16]. The number of lipid A acyl chains is directly correlated with the ability to induce cytokine production, whereas hexa-acylated forms usually have the greatest immunostimulating ability [17].

Gastrointestinal (GI) symptoms, including but not limited to, abdominal pain, diarrhea, and constipation, are frequently encountered in individuals with ASD [18]. However, there is no direct evidence showing the correlation between GI symptoms and ASD with respect to the endotoxin levels. Rather, it is believed that the gut plays a role in ASD etiology [19]. Many studies have demonstrated the presence of distinct microbiota between individuals with ASD and neurotypical control subjects [20,21,22,23]; although these studies have been performed exclusively in children, their results vary [24].

Owing to the conflicting data currently available, it is worth investigating immunological dysregulation, GI inflammation, and the role of endotoxins in the guts of neurotypical control subjects and children with ASD. Therefore, we categorized the microbiomes of control and ASD samples based on endotoxicity, and identified the bacterial species distributions and variations and the complex LPS metabolic pathways, with an emphasis on lipid A composition, which is responsible for most of the toxicity of LPSs.

## 2. Materials and Methods

### 2.1. Data Mining and Stratification

Using the Sequence Read Achieved (SRA) toolkit, all sample data from 36 individuals with ASD and 21 neurotypical control subjects labeled with the SRA number SRP182132 and Bio project number PRJNA516054 deposited in the Nataional Center of Biotechnology Information (NCBI) database (http://www.ncbi.nlm.nih.gov/Traces/sra, accessed on 10 June 2020) were mined. We used the Ribosomal Database Project (RDP) classifier option of RDP pipeline (Center for Microbioal Ecology, Michigan State University, USA. http://rdp.cme.msu.edu/classifier/classifier.jsp, accessed on 5 July 2020) from the online tool RDP for bacterial classification, using a confidence cutoff of 80 during submission. Analyzed data from a previous publication [23] were also compared and adopted for this analysis. Using R software v4.0 (R Foundation for Statistical Computing, Vienna, Austria. https://www.R-project.org/, accessed on 5 August 2020), we further stratified the control and ASD samples into different genera and species. The R package “tidyverse” was utilized to identify abundant bacteria, at both the genus and species level. A simple search helped to identify the genera and species status (Gram-negative or -positive).

### 2.2. Lipid A Production Pathway Selection

Pathway prediction is generally performed based on the presence of particular gene or protein homologues or specific enzymes. The Kyoto Encyclopedia of Genes and Genomes (KEGG) database (https://www.genome.jp/kegg/pathway.html, accessed on 17 June 2020) [25] is known for different pathway predictions; therefore, the LPS metabolic pathway was explored and generic lipid A disaccharide production was critically analyzed, filtered out, and schematically represented. Nine essential enzymes (LpxA, LpxC, LpxD, LpxH, LpxB, LpxK, WaaA, LpxL, and LpxM) involved in the lipid A synthesis pathway were considered.

### 2.3. Enzyme Selection and Prediction of End-Products

**Figure 1:**
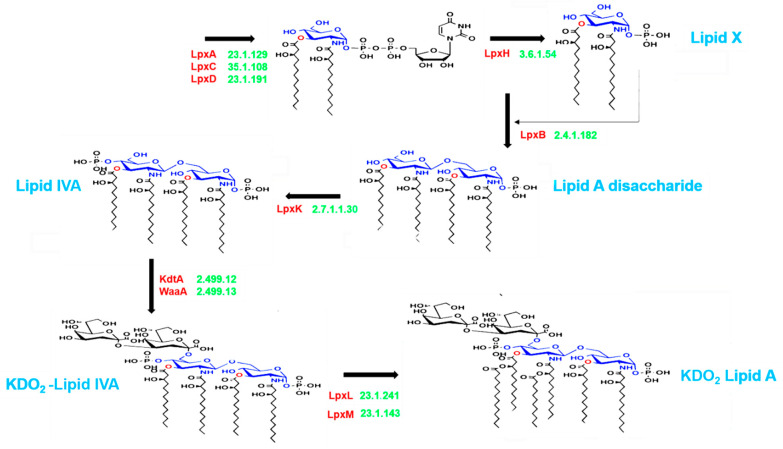
Schematic representation of lipopolysaccharide (LPS) biosynthesis, leading to the production of the inner part of the LPS (lipid A), as depicted in the complex pathway identified using the Kyoto Encyclopedia of Genes and Genomes (KEGG) database. The pathway corresponds to endotoxin production and involves nine essential enzymes (indicated in red), including rate-limiting enzymes. The Enzyme Commission numbers and products are in green and blue, respectively. Lipid X, 2,3-diacyl-GlcN-1-phosphate; Lipid IVA, lipid A disaccharide bisphosphate; KDO_2_, 2-keto-3-deoxy-D-mannooctanoic acid.

The lipid A domain of LPS is generated via nine conserved steps known as the Raetz pathway [26]. As shown in Figure 1, the first stage of synthesis involves the fatty acylation of uridine diphosphate (UDP)-N-acetylglucosamine by LpxA (Enzyme Commission (EC) 2.3.1.129). This is followed by deacetylation by LpxC (EC 3.5.1.108), as the committed step in lipid A disaccharide production. Following deacetylation, a second R-3-hydroxymyristate chain is added by LpxD (EC 2.3.1.191), to make UDP-2,3-diacyl-glucosamine (GlcN). This results in the production of a pyrophosphate linkage of UDP-2,3-diacyl-GlcN, which is later cleaved by LpxH (EC 3.6.1.54) to form 2,3-diacyl-GlcN-1-phosphate (lipid X). With lipid X, LpxB (EC 2.4.1.182) condenses UDP-2,3-diacyl-GlcN to generate disaccharide 1-phosphate, which is subsequently phosphorylated by LpxK (EC 2.7.1.130) to form lipid IVA (lipid A disaccharide bisphosphate). The two previously formed 2-keto-3-deoxy-D-mannooctanoic acid (KDO_2_) residues formed previously are then incorporated by the enzyme, WaaA (EC 2.499.12), while LpxL (EC 23.1.241) incorporates laurate. The last step of lipid A biosynthesis involves the addition of myristoyl residues to the distal glucosamine unit by LpxM (EC 23.1.243).

Using the UniProt database (https://www.uniprot.org/, accessed on 29 June 2020) [27], we searched for the presence of every enzyme in our identified genera. The percentage distributions were calculated and used for the construction of a heat map—comprising both control and ASD samples—using the R package “pheatmap”. To identify the distribution pattern of gut-associated genera, on the basis of the last enzymes predicted to be involved in their pathway for LPS metabolism, a Sankey-diagram was generated using the R package “NetworkD3“. This helped in predicting the end-product of gut bacteria during the lipid A synthesis.

## 3. Results

### 3.1. Data Stratification

**Figure 2:**
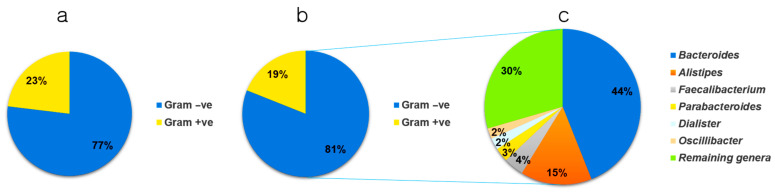
Pie chart depicting the stratification of microbiomes from (**a**) control and (**b**) autism spectrum disease (ASD) samples, based on Gram-positive/negative status, at the genus level. (**c**) Distribution of the six most abundant Gram-negative genera found in individuals with ASD. Gram +ve, Gram-positive; Gram –ve, Gram-negative.

Data obtained from 36 and 21 children with and without ASD, respectively, were carefully analyzed and stratified into two strata based on the results of Gram staining (genus and species level). In the control samples, 89 genera were identified, and based on the average value distribution (AVD) of the abundance of each genus, 23% were Gram-positive and 77% were Gram-negative (Figure 2a). However, in individuals with ASD, 108 genera were identified, and based on the AVD of their abundance, 19% were Gram-positive and 81% were Gram-negative (Figure 2b). This indicated a higher number of Gram-negative bacteria in individuals with ASD (5.19%). The results presented in Figure 2c show the six most abundant genera and their percentage distributions in individuals with ASD. These included *Bacteroides* (44%), *Alistipes* (15%), *Faecalibacterium* (4%), *Parabacteroides* (3%), *Dialister* (2%), and *Oscillibacter* (2%). The remaining genera occupied ~30% of the total number of bacteria.

### 3.2. Microbial Distribution among Children with ASD at the Genus and Species Level

**Figure 3:**
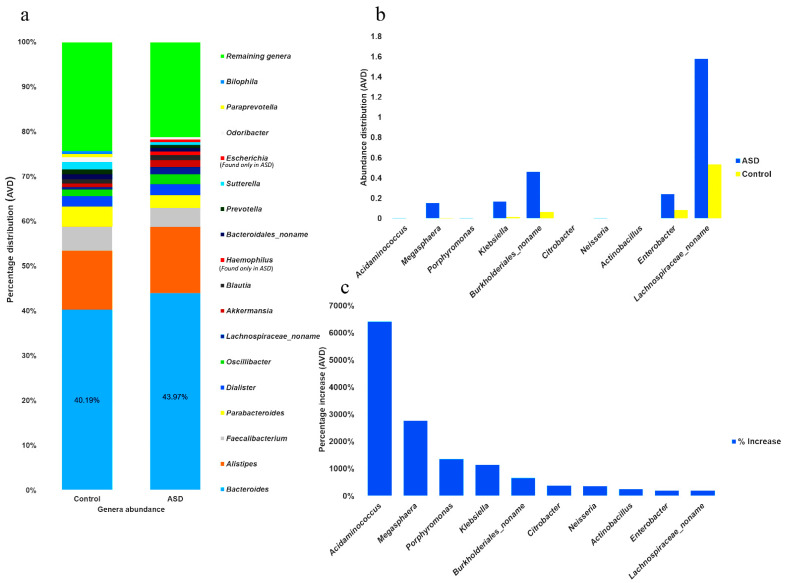
Differential distribution of the gut microbiome at the genus level. (**a**) The 15 most abundant Gram-negative genera in the control and Autism Spectrum Disorder (ASD) samples. (**b**) Bar chart showing the average distribution (AVD) of the 10 genera with the greatest increases in abundance in individuals with ASD and control subjects. (**c**) Bar chart indicating the percentage increase of the 10 genera with the greatest increase in abundance.

The abundance of all genera was determined in individuals with ASD and control subjects, and the 15 most abundant genera were identified. In individuals with ASD, the most abundant genera (MAG) constituted approximately 78% of the AVD of the total Gram-negative population (Figure 3a) and comprised (from high to low) *Bacteroides*, *Alistipes*, *Faecalibacterium*, *Parabacteroides*, *Dialister*, *Oscillibacter*, *achnospiraceae_noname*, *Akkermansia*, *Blautia*, *Haemophilus*, *Bacteroidales_noname*, *Prevotella*, *Sutterella*, *Escherichia*, and *Odoribacter*. The 15 most abundant genera in the control subjects (MAG0), which constituted approximately 75% of the total population, were identified as *Bacteroides*, *Alistipes*, *Faecalibacterium*, *Parabacteroides*, *Dialister*, *Sutterella*, *Oscillibacter*, *Bacteroidales_noname*, *Odoribacter*, *Prevotella*, *Blautia*, *Akkermansia*, *Parabacteroides*, *Bilophila*, and *Lachnospiraceae_noname*. The five MAG were the same in both groups; however, the abundance of *Bacteroides* in individuals with ASD was 9.4% higher than the AVD of that in the control samples. Furthermore, *Haemophilus* and *Escherichia*, two genera shown to be significantly more abundant in individuals with ASD, were either completely absent or significantly less abundant in the control subjects, as shown in Figure 3a and Supplementary Tables S1 and S2.

Moreover, the difference in genera distribution between the control and ASD samples, based on the percentage increase in ASD samples, revealed that the 10 most increased genera (MIG) were *Acidaminococcus*, *Megashphaera*, *Porphyromonas*, *Klebsiella*, *Burkholderiales_noname*, *Citrobacter*, *Neisseria*, *Actinobacillus*, *Enterobacter*, and *Lachnospiraceae_noname* (Figure 3b and Supplementary Table S3). Among those MIG, *Acidaminococcus, Megashphaera*, and *Porphyromonas* showed the highest percentage increase in individuals with ASD (Figure 3c). However, newly identified genera, such as *Acidaminococcaceae_unclassified*, *Morganella*, *Cardiobacteriaceae_unclassified*, *Bacteroidetes_noname*, and *Alloprevotella* were completely absent in control samples.

At the species level, we filtered out the 20 most abundant species, of which *Bacteroides uniformis* appeared to be the most abundant, with an average distribution of 9.11% among individuals with ASD. This was followed by *Bacteroides vulgatus*, with an average distribution of 6.4%, which was similar to that in control samples in terms of the total AVD (Supplementary Table S4). Some important species, such as *Akkermansia muciniphila*, were absent in the control samples but present at significant levels in individuals with ASD. Furthermore, the distribution change at the species level revealed 20 Gram-negative species with the greatest increase in individuals with ASD. *Klebsiella oxytoca* showed the greatest increase (6253%), followed by *Bacteroides clarus* (3020%). Although *Bacteroides fragilis* was the 20th most increased species (52%), it was found to have the highest average value (2.11%) among the 20 species that showed increased abundance in individuals with ASD (Supplementary Table S5).

### 3.3. Gut-Microbial Metabolic Pathway

**Figure 4:**
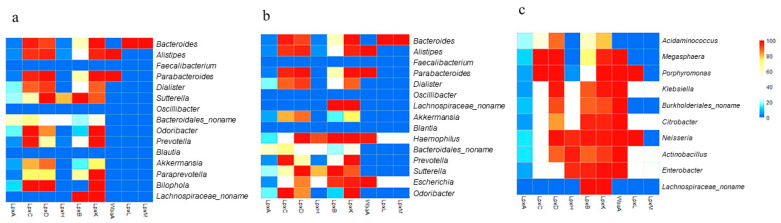
Heat map showing the percentage distributions of strains harboring each of the nine essential lipopolysaccharide (LPS) synthesis enzymes in the 15 most abundant genera (MAG) in control samples (**a**), the 15 MAG in ASD samples (**b**), and the 10 genera with the greatest increases in abundance (**c**), as determined by analyzing bacterial genome sequences. Each cell represents the percentage of strains predicted to harbor a particular enzyme. Red indicates 100% availability, while blue indicates 0%.

LPS metabolism (conserved in most Gram-negative bacteria) was selected and analyzed. The percentage of available strains at the genus level possessing each enzyme extracted from the UniProt database are displayed in Supplementary Tables S1–S3. With respect to LPS end products, the genera that possess a complete set of enzymes would have high potential for producing LPS end products. In the control samples, UniProt analysis revealed that the majority of the MAG in both the control and ASD samples utilized the early-stage enzymes LpxA, LpxC, LpxD, LpxB, and LpxK (Figure 1), with a large number of available strains. However, a greater number of genera in individuals with ASD possessed LpxH and produced lipid X. Furthermore, in control samples, only one of the 15 MAG possessed all enzymes up to the last enzyme in the process, LpxM, while 3 of the 15 MAG possessed these enzymes in the ASD samples (Figure 4a,b). *Bacteroides*, the genus that showed great increase in abundance in ASD samples (Figure 3a), possessed a complete set of enzymes with the ability to produce LPS end products. Two genera that have recently been shown to be important in ASD, *Haemophilus* and *Escherichia*, were not only significantly more abundant in ASD samples, but also possessed a complete set of enzymes with the potential to produce LPS end products (Figure 4b).

Next, the availability of LPS synthesis enzymes was analyzed for the 10 MIG in ASD samples (Figure 4c). All genera possessed a reasonable percentage of LpxA, LpxC, LpxD, LpxB, and LpxK, and interestingly, the majority of genera possessed the late-stage enzymes, LpxH, WaaA, and LpxL, with a reasonable number of available strains. This may imply relatively greater lipid A production in individuals with ASD. Furthermore, four genera (*Klebsiella, Citrobacter, Actinobacillus*, and *Enterobacter*) possessed a complete set of enzymes and therefore, have high potential for producing LPS end products. This evidence suggested that individuals with ASD have a greater potential to produce LPSs than do non-ASD individuals.

### 3.4. Lipid A Synthesis Stages and Gram-Negative Bacterial Distribution in Individuals with ASD

**Figure 5:**
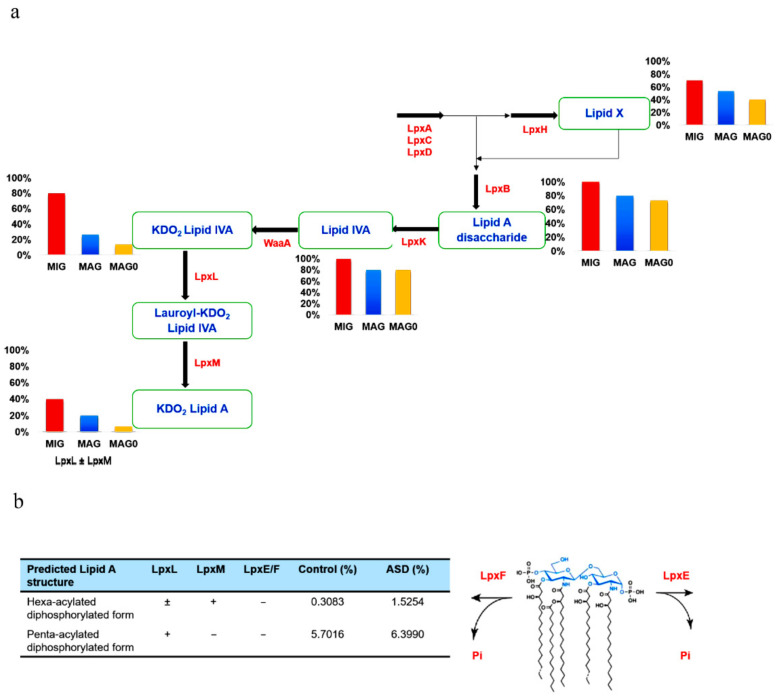
(**a**) Prediction of lipopolysaccharide (LPS) products based on gut bacteria counts at the genus level. The 10 most increased genera (MIG) and the 15 most abundant genera for both autism spectrum disorder (ASD) (MAG) and control subjects (MAG0) were considered. The products of enzymes are written in blue. The bar plot depicting the genera count at each stage is below or behind the respective enzyme product. The percentage value indicates the proportion of genera capable of producing the particular metabolic product at each respective stage. (**b**) Predicted distribution of the late-stage lipid A products at the species level. Key enzymes for the lipid A acylation and modification were analyzed at the species level and their relative abundances were calculated in the ASD and control samples. Lipid X, 2,3-diacyl-GlcN-1-phosphate; Lipid IVA, lipid A disaccharide bisphosphate; KDO_2_, 2-keto-3-deoxy-D-mannooctanoic acid.

We further categorized the data on the basis of lipid A synthesis stages, with respect to the enzymes involved. The summary of each stage of lipid A synthesis, as depicted in the KEGG pathway in relation to our data, is displayed in Figure 5a. This showcases the genera distributions with respect to each available enzyme among the 15 most abundant genera in individuals with ASD (MAG) and control subjects (MAG0), as well as the 10 MIG. We compared the results of the MIG group with those of the MAG and MAG0 groups. We can deduce that the MIG and MAG groups contained more LpxH-possessing genera than did the MAG0, and therefore, more genera that can produce lipid X. Furthermore, the MIG and MAG groups showed a greater potential for producing the last-stage products of lipid A, as they possessed a complete set of enzymes up to LpxL and LpxM (Figure 5a).

With respect to the late-step lipid A biosynthesis, LpxL acyltransferase is required for the penta-acylation of lipid A as in *Burkholderia cenocepacia* [28] and LpxM is needed for the hexa-acylation of lipid A, such as in *E. coli* [29]. Furthermore, two bacterial enzymes, LpxE and LpxF, catalyze the removal of phosphate groups from lipid A to yield a dephosphorylated form predicted to be less immunogenic (Figure 5b) [30]. To obtain more accurate prediction results, we performed further analysis at the species level. Key enzymes responsible for the late-stage acylation and modification were analyzed at the species level and their relative abundances were calculated in the gut microbiome. The proportions of both penta- and hexa-acylated forms is predicted to increase in individuals with ASD compared to those in the control sample. In particular, given that the hexa-acyl lipid A with two phosphates is a potent immunostimulant, the percentage of this unmodified *E. coli* lipid A molecule is likely to increase significantly in individuals with ASD.

**Figure 6:**
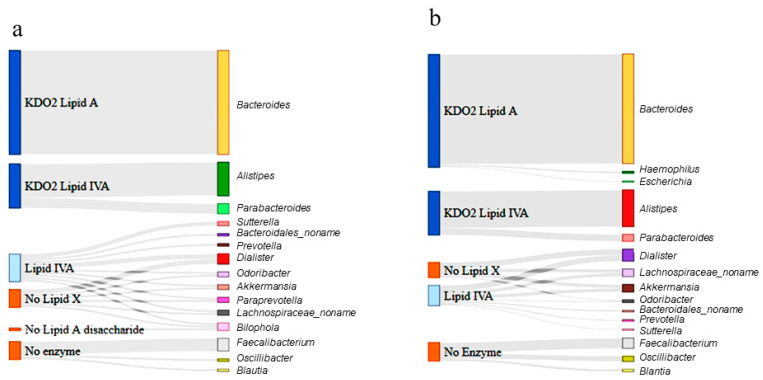
Sankey diagram depicting the lipopolysaccharide (LPS) metabolism pathway in the 15 most abundant genera (MAG) in the control (**a**) and ASD samples (**b**). The genera differentially enriched in ASD children are shown in the right bars as targets. The respective enzymatic stage and product for each genus is shown in the left bars as the source. The Sankey diagram depicts the entire pathways for differentially categorized genera. The width of the enzyme–genus association represents the abundance of the genus. Lipid X, 2,3-diacyl-GlcN-1-phosphate; Lipid IVA, lipid A disaccharide bisphosphate; KDO_2_, 2-keto-3-deoxy-D-mannooctanoic acid.

Next, Sankey diagrams were constructed to summarize the distribution of each genera derived from the raw data and to present the metabolic product of each enzyme shared among the respective groups at the genus level (Figure 6). In the control group (Figure 6a), *Bacteroides*, the most abundant genus, could produce a KDO_2_ lipid A, as it harbored the final synthesis enzyme, LpxM, whereas *Alistipes* and *Parabacteroides* could only produce KDO_2_ lipid IVA. However, the majority of the less abundant genera, including *Dialister* and *Akkermansia*, could produce up to lipid IVA and a reasonable number of genera, including *Odoribacter*, bypassed lipid X production. Three genera (*Faecalibacterium*, *Oscillibacter*, and *Blautia*) possessed no enzymes in the lipid A synthesis pathway.

A similar categorization was performed for the ASD samples. The Sankey diagram presented in Figure 6b shows that the gut bacteria in the ASD samples were more likely to produce the final-stage products than were those in the control samples. Based on the abundance of each genus, it was predicted that the final-stage products (KDO_2_ lipid A and KDO_2_ lipid IVA) comprised 68% of the total products when genera without enzymes were excluded. Overall, the gut bacteria of individuals with ASD were predicted to produce 5.2% more LPS end-products than those of the control subjects.

## 4. Discussion

In recent decades, there have been speculations regarding persistent links between microbiomes and brain disorders, which have now been established at a certain level. As the inception of these links, a potential link between the microbiome and ASD was confirmed by Finegold et al. [31], who observed differences in gut bacteria between ASD children and control subjects Subsequently, many related findings have been reported, especially regarding GI dysfunction in individuals with ASD [32,33,34]. In addition to GI dysfunction, immune dysregulation is also an essential factor with a high prevalence in individuals with ASD [35,36]. While many studies have highlighted the relationship between gut bacteria, the GI tract, immune dysregulation, and ASD, a direct contribution by the microbiota towards immune dysregulation requires elucidation.

Based on in silico analysis, this study focused on these two major factors and provided a catalog of gut bacteria in individuals with ASD and control subjects, with respect to their Gram-negative status, which is closely related to bacterial endotoxicity and rate of LPS production. Our data showed a marked increase in the prevalence of Gram-negative bacteria in individuals with ASD compared to control subjects, with a 5.19% increase in the AVD. This may indicate greater LPS production and immune dysregulation in individuals with ASD, which is consistent with previous findings in ASD [37] and Alzheimer’s disease [38] patients. Our results appear to be connected with the previously reported increased serum LPS levels and impaired behavioral scores in individuals with ASD [39].

The 10 MIG were identified based on a comparison with their abundance in the control subjects. Although the relative abundance of the MIG was low, most possessed more complete sets of lipid A biosynthesis enzymes than did the MAG and MAG0 groups, possibly producing a greater amount of lipid A end products. *Acidaminococcus* was found to have the highest fold increase (6409.81%) in individuals with ASD, which is in accordance with previous findings [40]. In addition, the genus with the third highest increase, *Porphyromonas*, is reported to have highly heterogeneous LPSs, accommodating more lipid A species and possessing the ability to activate the important innate immune receptors TLR1, TLR2, TLR2, and TLR4 [41]. Collectively, our results indicated that these differentially abundant genera may be responsible for the GI symptoms of individuals with ASD.

Our definitive finding at the species level revealed that *Bacteroides fragilis* was the most abundant among the 20 species with an increased abundance in individuals with ASD. A recent human GI microbiome study showed an abundance of *Bacteroides fragilis*, which generates LPSs [42], possibly contributing to neuroinflammation. It is also noteworthy that LPS from *Alistipes* spp. is considered highly inflammatory, resulting in an increase in the number of Th_17_ cells [43,44]. This further implicates *Alistipes* spp. as gut bacteria with emerging importance in inflammation and mental health.

Based on our findings, almost all the bacteria categorized at the genus level contained LpxA, LpxC, LpxD, LpxB, and LpxK. The lipid A immunostimulating principles indicate that the final-stage products are more immunogenic and more important for the immune response than the early-stage products. In this regard, the MAG in ASD samples contained more synthesis enzymes than did their counterparts in the control samples. Further, from our findings, the MIG group possessed more late-stage enzymes than did the MAG group, implying that the synthesis of lipid A end products is more likely to increase in individuals with ASD. Considering the notion that the chemical structure of LPS can vary considerably among different species [17], we analyzed the presence of late-stage enzymes at the species level. Species-level analysis also indicated that the proportion of the unmodified lipid A containing six acyl chains and two phosphates is likely to increase in individuals with ASD (Figure 5b), which supports our findings at the genus level.

Emiola et al. [45] reported that decreasing LpxH inhibition increases the rate of Lipid A production and also increases the LpxM copy number, which equally contribute toward the greater production of lipid A end products. This finding is in accordance with our results, considering that more LpxM- and LpxH-possessing genera were found in the MIG group than in the MAG and MAG0 groups, and a greater AVD of the MAG was shown to produce the final products. Overall, we propose that more immunogenic LPS could be produced in individuals with ASD, which may be related to their well-acknowledged GI problems and elevated inflammatory conditions.

## 5. Conclusions

We aimed to explore the channels of communication between gut bacteria, LPS metabolism, and neurological inflammation in individuals with ASD. To the best of our knowledge, this is the first in silico analysis of bacterial genome and gut microbiome data from individuals with ASD to focus on LPS distribution. Our findings showed that dysbiosis of gut microbiomes may correlate with neuronal inflammation and abnormalities in individuals with ASD. Moreover, as a potential risk factor, our results suggested that the Gram-negative bacteria that are increased in the gut of individuals with ASD, especially those possessing a complete set of lipid A synthesis enzymes, may be novel targets for the treatment of ASD and other neurodegenerative disorders.

## Data Availability

Data are available from the authors upon reasonable request.

## Supplementary Materials

The following are available online at https://www.mdpi.com/2072-6643/13/2/688/s1: Table S1: Most abundant genera in control (MAG0) and their % distribution; Table S2: Most abundant genera in ASD (MAG) and their % distribution; Table S3: Most increased genera and their % distribution; Table S4: Most abundant species in control and individuals with ASD; Table S5: Most increased species and their % distribution.
