# Supplementary material for: Gut Microbiome-Based Analysis of Lipid A Biosynthesis in Individuals with Autism Spectrum Disorder: An In Silico Evaluation
Source: Nutrients. 2021 Feb 21;13(2):688. doi: 10.3390/nu13020688 (PMC7924848; doi:10.3390/nu13020688)
Supplement: Supplementary file 1 [file nutrients-13-00688-s001.pdf]

Supplementary Table S1. Most abundant genera in control (MAG0) and their strains % distribution.

Genus	AVD	LpxA	LpxC	LpxD	LpxH	LpxB	LpxK	WaaA	LpxL	LpxM
Bacteroides	40.1988	2.39	96.06	91.96	2.38	62.68	98.26	0	100	100
Alistipes	13.1825	3.08	93.02	95.45	4.26	48.89	95.35	100	0	0
Faecalibacterium	5.4047	0	0	0	0	0	0	0	0	0
Parabacteroides	4.4801	2.71	96	98.28	0.6	64.41	98.31	100	0	0
Dialister	2.3337	17.65	88.24	92.86	0	40	88.24	0	0	0
Sutterella	1.6737	19.79	64.52	100	80.95	100	89.47	0	0	0
Oscillibacter	1.4705	0	0	0	0	0	0	0	0	0
Bacteroidales_noname	1.1350	61.52	69.64	37.5	44.44	19.35	56.9	0	0	0
Odoribacter	1.0652	16.67	100	84.62	0	11.11	100	0	0	0
Prevotella	1.0291	4.57	97.33	65.55	2.52	54.55	98.51	0	0	0
Blautia	0.9643	0	0	0	0	0	0	0	0	0
Akkermansia	0.8059	6.49	81.25	87.5	0	15.79	75	0	0	0
Paraprevotella	0.7158	2.44	75	75	0	66.67	100	0	0	0
Bilophila	0.6073	12.5	100	100	0	0	100	0	0	0
Lachnospiraceae_noname	0.5326	0.67	0	0	0	100	100	0	0	0

Abbreviations: AVD, average value distribution.

Supplementary Table S2. Most abundant genera in ASD (MAG) and their strains % distribution.

Genus	AVD	LpxA	LpxC	LpxD	LpxH	LpxB	LpxK	WaaA	LpxL	LpxM
Bacteroides	43.9726	2.39	96.06	91.96	2.38	62.68	98.26	0	100	100
Alistipes	14.7629	3.08	93.02	95.45	4.26	48.89	95.35	100	0	0
Faecalibacterium	4.2211	0	0	0	0	0	0	0	0	0
Parabacteroides	2.8816	2.71	96	98.28	0.6	64.41	98.31	100	0	0
Dialister	2.4613	17.65	88.24	92.86	0	40	88.24	0	0	0
Oscillibacter	2.1972	0	0	0	0	0	0	0	0	0
Lachnospiraceae_noname	1.5793	0.67	0	0	0	100	100	0	0	0
Akkermansia	1.5705	6.49	81.25	87.5	0	15.79	75	0	0	0
Blautia	1.1117	0	0	0	0	0	0	0	0	0
Haemophilus	0.8035	14.75	51.19	98.92	90.48	99.04	99	96.88	49.18	50.83
Bacteroidales_noname	0.7764	61.52	69.64	37.5	44.44	19.35	56.9	0	0	0
Prevotella	0.6930	4.57	97.33	65.55	2.52	54.55	98.51	0	0	0
Sutterella	0.5924	19.79	64.52	100	80.95	100	89.47	0	0	0
Escherichia	0.5787	4.91	43.43	85.53	49.5	93.91	96.42	100	31.18	43.63
Odoribacter	0.5664	16.67	100	84.62	0	11.11	100	0	0	0

Abbreviations: AVD, average value distribution; ASD, autism spectrum disorder.

Supplementary Table S3. Most increased genera (MIG) and their strains % distribution.

Genus	AVD-CN	AVD-ASD	%Increase	LpxA	LpxC	LpxD	LpxH	LpxB	LpxK	WaaA	LpxL	LpxM
Acidaminococcus	8.86E-05	0.0057	6409.81	19.05	57.14	87.5	0	62.5	80	0	0	0
Megasphaera	0.0053	0.1523	2774.09	11.11	100	100	0	71.43	95.45	100	0	0
Porphyromonas	0.0002	0.0042	1352.23	6.93	100	100	4.35	34.88	100	100	100	0
Klebsiella	0.0134	0.1672	1144.98	5.56	49.21	97.73	50.56	89.94	97.18	100	45.8	27.08
Burkholderiales_noname	0.0608	0.4617	658.58	9.51	46.46	90.91	34.88	88.46	90.2	100	0	0
Citrobacter	0.0007	0.0034	383.44	5.45	47.52	83.33	52.94	95.08	95.95	100	31.38	34.44
Neisseria	0.0008	0.0039	354.85	13.03	48.42	98.11	94.23	97.12	97.98	100	100	0
Actinobacillus	0.0004	0.0017	242.33	13.44	52.08	88.46	96.15	88.89	92.31	100	51.02	51.06
Enterobacter	0.0802	0.2407	199.98	6.53	48.35	52	95	95.2	98.61	100	44.16	25.78
Lachnospiraceae_noname	0.5326	1.5793	196.52	0.67	0	0	0	100	100	0	0	0

Abbreviations: AVD, average value distribution; ASD, autism spectrum disorder; CN, control.

Supplementary Table S4. Most abundant species in control and individuals with ASD.

SPECIES	AVD-CN	SPECIES	AVD-ASD
Bacteroides uniformis	8.2699	*Bacteroides uniformis*	9.1107
Alistipes putredinis	6.7517	*Bacteroides vulgatus*	6.3990
Bacteroides vulgatus	5.7016	*Alistipes onderdonkii*	6.0277
Faecalibacterium prausnitzii	5.4047	*Alistipes putredinis*	5.0361

Bacteroides dorei	3.9207	*Bacteroides ovatus*	4.4489
Bacteroides ovatus	3.3417	*Faecalibacterium prausnitzii*	4.2212
Bacteroides massiliensis	3.3369	*Bacteroides cellulosilyticus*	3.8806
Bacteroides caccae	3.0294	*Bacteroides dorei*	2.9137
Bacteroides plebeius	2.8978	*Bacteroides caccae*	2.7306
Alistipes onderdonkii	2.7979	*Dialister invisus*	2.4613
Barnesiella intestinihominis	2.5968	*Bacteroides massiliensis*	2.1281
Parabacteroides merdae	2.2584	*Bacteroides fragilis*	2.1123
Dialister invisus	2.1834	*Bacteroides stercoris*	2.1052
Bacteroides cellulosilyticus	1.8241	*Alistipes finegoldii*	1.5904
Sutterella wadsworthensis	1.6737	*Akkermansia muciniphila*	1.5705
Alistipes shahii	1.5342	*Bacteroides thetaiotaomicron*	1.5166
Bacteroides fragilis	1.3903	*Alistipes shahii*	1.3599
Bacteroides thetaiotaomicron	1.3552	*Barnesiella intestinihominis*	1.3320
Bacteroides stercoris	1.1828	*Parabacteroides merdae*	1.1630

Abbreviations: AVD, average value distribution; ASD, autism spectrum disorder; CN, control.

Supplementary Table S5. Most increased species and their strains % distribution.

Species	AVD-CN	AVD-ASD	% Increase	LpxA	LpxC	LpxD	LpxH	LpxB	LpxK	WaaA	LpxL	LpxM
Klebsiella oxytoca	0.0005	0.0377	6253	6.91	40.91	90.91	51.85	92.86	100	100	32.43	35.14
Bacteroides clarus	0.0205	0.6414	3020	1.67	100	100	0	33.33	100	0	0	0
Acidaminococcus fermentans	8.86E-05	0.0026	2931	21.43	66.67	66.67	0	66.67	66.67	0	0	0
Escherichia unclassified	0.0027	0.0558	1964	5.2	46.58	86.75	51.16	97.23	100	100	32.38	51.16
Bacteroides intestinalis	0.0480	0.6447	1243	1.41	1	1	0	47.06	85.71	0	0	0
Klebsiella pneumoniae	0.0125	0.1259	904	7.21	68.35	98.7	63.56	92.22	100	92.11	50.27	28.48
Bacteroides faecis	0.0770	0.7648	892	3.7	100	100	0	33.33	60	0	0	0
Citrobacter freundii	0.0003	0.0024	602	8.57	36	87.5	50	48.39	92.5	100	56.9	64.91
Haemophilus sputorum	0.0031	0.0107	245	16.67	50	100	100	100	100	0	50	50
Desulfovibrio piger	0.0179	0.0550	207	0	0	0	0	0	0	0	0	0
Oxalobacter formigenes	0.0014	0.0042	202	10	50	100	100	100	100	0	0	0
Alistipes unclassified	0.1135	0.3160	178	3.08	93.02	95.45	4.26	48.89	95.35	100	0	0
Phascolarctobacterium succinatutens	0.2166	0.5515	155	15.79	100	100	0	100	100	0	0	0
Akkermansia muciniphila	0.8059	1.5705	95	6.61	100	90	0	18.18	83.33	0	0	0
Bacteroides stercoris	1.1828	2.1052	78	3.7	100	100	5.88	85.71	92.86	100	0	0
Bilophila wadsworthia	0.0333	0.0559	68	12.5	100	100	0	100	100	0	0	0
Alistipes senegalensis	0.0197	0.0327	66	0	0	0	0	0	0	0	0	0
Bilophila unclassified	0.1884	0.2985	58	12.5	100	100	0	0	100	0	0	0
Bacteroides coprocola	0.0460	0.0706	54	3.57	100	100	0	66.67	100	100	0	0
Bacteroides fragilis	1.3903	2.1123	52	0.94	100	96.15	2	81.58	100	100	0	0

Abbreviations: AVD, average value distribution; ASD, autism spectrum disorder; CN, control.
